# A Selective Chromogenic Plate, YECA, for the Detection of Pathogenic *Yersinia enterocolitica*: Specificity, Sensitivity, and Capacity to Detect Pathogenic *Y. enterocolitica* from Pig Tonsils

**DOI:** 10.4061/2011/296275

**Published:** 2011-06-09

**Authors:** M. Denis, E. Houard, A. Labbé, M. Fondrevez, G. Salvat

**Affiliations:** Unité Hygiène et Qualité des Produits Avicoles et Porcins, Anses, BP 53, 22440 Ploufragan, France

## Abstract

A new selective chromogenic plate, YECA, was tested for its specificity, sensitivity, and accuracy to detect pathogenic *Y. enterocolitica* from pig tonsils. We tested a panel of 26 bacterial strains on YECA and compared it to PCA, CIN, and YeCM media. Detection of pathogenic *Y. enterocolitica* was carried out on 50 pig tonsils collected in one slaughter house. Enrichment was done in PSB and ITC broths. Streaking on YECA and CIN was done in direct, after 24H incubation of ITC, after 48H incubation of PSB and ITC. All the plates were incubated at 30°C during 24 hours. Presence of typical colonies on CIN and YECA was checked, and isolates were biotyped. Pathogenic *Y. enterocolitica* strains showed an important growth on YECA with small and red fuchsia colonies while biotype 1A exhibited very few violet colonies. Enrichment in ITC during 48H gave the best performance for detecting positive samples in pathogenic *Y. enterocolitica*, and YECA could detect directly pathogenic *Y. enterocolitica* strains (2, 3, and 4). Use of YECA in combination with ITC generates a time-saver by giving a positive test in 72H.

## 1. Introduction

*Y. enterocolitica* is a common cause of acute enteritis in temperate and cold countries worldwide, including France. The main symptoms of human yersiniosis are diarrhea, fever, and abdominal pain. Bacteria usually remain in the intestinal tract, but may also invade their host, causing abscesses in deep organs and septicemia in patients with underlying conditions [1]. 

In 2009, yersiniosis was, for the sixth consecutive year, the third most frequently reported human zoonosis in the Europe, with a total of 8,354 confirmed cases [2]. *Y. enterocolitica* was the most common *Yersinia* species reported in human cases in European countries, accounting for 93.8% of all confirmed cases of yersiniosis [3].

Pathogenic *Y. enterocolitica* strains belong to biotypes 1B, 2, 3, 4, and 5, whereas biotype 1A strains are nonpathogenic and widespread in the environment [4]. In France and most other countries worldwide, biotype 4 is the most prevalent biotype isolated from humans (69%), followed by biotype 2 (30%) and biotype 3 (1%) [1]. 

Human infections most frequently occur as sporadic cases or small family-centered outbreaks [1]. *Y. enterocolitica *is transmitted by the fecal-oral route, and its principal reservoirs are animals. Pigs are considered the principal reservoir for the types of *Y. enterocolitica* pathogenic to humans, although other animal species, such as cattle, sheep, poultry, fish, deer, small rodents, cats, and dogs, may also carry pathogenic biotypes [4–9]. Contaminated drinking water is also reported as source of biotype 1B *Yersinia* infection [10]. 

The incidence of yersiniosis due to pork consumption in humans was recently estimated at 2.8 cases per 100,000 inhabitants per year in Europe [11]. This bacterium is the second most frequent contaminant of pig products, after *Salmonella* (3.3) and ahead of *Campylobacter* (2.1). Pigs do not develop clinical signs, but they do carry *Y. enterocolitica* in their oral cavity, on tongues and tonsils, and in lymph nodes, and excrete this bacterium in their feces [12, 13]. Bioserotype 4/O: 3 is the most prevalent pathogenic bioserotype isolated from pigs [14–20]. 

Detection of *Yersiniosis* is carried out by using ISO 10273-2003 method [21]. This method is recommended for both food and pig tonsil analyses [22] but involves time-consuming enrichment steps followed by plating on selective media [23]. This method involves enrichment in two broths, peptone sorbitol bile (PSB) broth, and irgasan-ticarcillin-potassium chlorate (ITC) broth, followed by a streaking on two plates, cefsulodin-irgasan-novobiocin (CIN) agar plateand *Salmonella-Shigella*-sodium deoxycholate-calcium chloride (SSDC) agar plate, respectively. Moreover, incubation of PSB broth can take up to five days. Recently, authors proposed modifications of the method in order to simplify the detection of *Y. enterocolitica*. Van Damme et al. (2010) [20] showed that the use of a two-day incubation period at 25°C, instead of five days, for the PSB broth resulted in a significantly higher recovery rate of *Yersinia*. Fondrevez et al. (2010) [24] demonstrated that streaking onto a CIN agar plate from ITC broth, recovered a larger number of positive samples than the ISO method. In addition, Weagant (2008) [25] has developed a chromogenic medium, *Yersinia enterocolitica* chromogenic medium (YeCM), for the specific detection of pathogenic *Y. enterocolitica*. However, difficulties were encountered to isolate pathogenic *Y. enterocolitica* colonies among the non-*Y. enterocolitica* colonies when using YeCM just after the enrichment step. It is the reason why a method involving streaking from ITC broth onto a CIN agar plate, followed by the streaking of typical *Y. enterocolitica* colonies onto the chromogenic medium, YeCM, was proposed by Fondrevez et al. (2010) [24]. This method allowed separation of *Y. enterocolitica *strains which carried pathogenic biotypes (red bull's-eye-like on YeCM) from the nonpathogenic biotype, 1A (blue-purple on YeCM) but an additional step of 24 hours is then needed.

Other alternative methods using PCR [8, 26] for detecting *Yersinia enterocolitica* from food or tonsil have been published. While PCR can be useful to quickly detect suspected positive samples, only culture method enable to recover isolates. 

In this work, we tested a new selective chromogenic plate, YECA, for its specificity and sensitivity. We tested its accuracy to detect pathogenic *Y. enterocolitica *from pig tonsils as *Y. enterocolitica *becomes a preoccupation in Europe's pig production. 

## 2. Materials and Methods

### 2.1. YECA: Yersinia Enterocolitica Agar—Selective Chromogenic Medium for Pathogenic Yersinia enterocolitica Screening

YECA developed by AES Chemunex (Combourg, France) is described as a chromogenic plate which permits to isolate specifically pathogenic *Yersinia enterocolitica; *the typical colonies are small and red fuchsia. This coloration is due to the presence of colour indicator revealed by sugar fermentation. The presence of desoxycholate improves the red fuchsia coloration of the pathogenic *Y. enterocolitica *colonies. The chromogenic substrate and tryptophan in the media allow the differentiation of pathogenic *Y. enterocolitica* strains from the nonpathogenic *Y. enterocolitica* strains (biotype 1A) and a majority of enterobacteria. 

### 2.2. Specificity of YECA

The specificity of YECA was tested against 26 strains listed in Table 1. These strains were *Yersinia enterocolitica*, *Yersinia*-like, and non-*Yersinia*. The following strains, *Morganella morganii, Pseudomonas sp.*, and *Serratia liquefaciens, *were obtained from nontypical colonies isolated from CIN after pig tonsil swab enrichment in ITC during the study of Fondrevez et al. (2010) [24]. Each strain was cultured in 5 mL of appropriated broth and incubation temperature for 24 hours. 

The cultures were all adjusted to 4 McFarland, corresponding to a concentration of 10^8^ to 10^9^ cells per mL. Streaking was then performed (1) on CIN agar plate (*Yersinia* Selective Agar Base and *Yersinia* Selective Supplement, Oxoid, Basingstoke, UK), (2) on YeCM medium (prepared in the laboratory as described by Weagant [25] and, (3) on YECA (AES chemunex, Combourg, France).

We measure the specificity by screening if the expected results for the *Yersinia enterocolitica* strains were obtained, that is, small and smooth colonies, with a red centre and a translucent rim, on CIN, red bull's-eye-like colonies for pathogenic *Y. enterocolitica* and blue-purple colonies for the nonpathogenic *Y. enterocolitica *on YeCM, small (<1 mm) red fuchsia colonies for pathogenic *Y. enterocolitica* and small (<1 mm) violet colonies for the nonpathogenic *Y. enterocolitica *on YECA. Moreover, if growth of bacteria was observed on plate, we noted the importance of growth in a scale from 1 to 5; 1 was applied when we observed one to 5 colonies on the plate, 5 when colonies covered all the plate.

### 2.3. Sensitivity of YECA

*Yersinia enterocolitica* strains from biotype 1A (IP124), 2 (IP383), 3 (IP29228), and 4 (IP134) (purchased from Pasteur Institute, Paris, France) were incubated in 5 mL of Brain Heart Infusion (BHI, AES Chemunex, Combourg, France) broth during 24 h at 30°C. The overnight cultures were all adjusted to 4 Mc Farland corresponding to a concentration of 10^8^ to 10^9^ cells per mL. For each biotype, a tenfold dilution was then done in tryptone salt. Then 100 *μ*L of the −5 to −10 dilutions were spread on PCA, CIN and YeCM plates, and 100 *μ*L of the −1 to −10 dilutions were spread on YECA plates. All the plates were incubated at 30°C for 24 hours and enumeration of the colonies was then performed. 

## 3. Detection of Pathogenic *Yersinia enterocolitica* from Pig Tonsils

**Figure 1:**
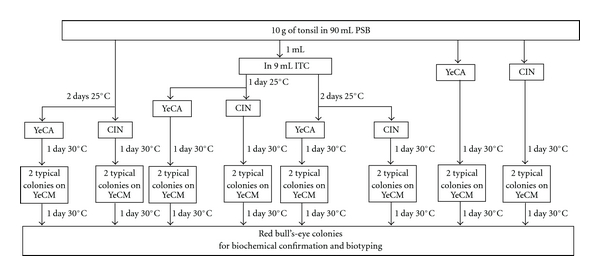
Overview of the methods used to isolate pathogenic *Yersinia enterocolitica* from pig tonsil in this study.

The assay was carried out on 50 pig tonsils collected from a slaughterhouse in five times (10 tonsils per visit), and culture method used has been presented in Figure 1. From each tonsil, 10 g were cut in small pieces and put into a bag containing 90 mL of PSB broth (prepared in the laboratory, as described in the ISO 10273:2003 method). After stomaching, 10 *μ*L were streaked directly onto YECA and CIN plates, and 1 mL was transferred in 9 mL of ITC broth. PSB and ITC were incubated at 25°C for 48 hours, before a second streaking onto YECA, and CIN. In addition, after 24 hours of enrichment in ITC broth, an extra streaking on YECA and CIN was performed. All the plates were incubated at 30°C for 24 hours.

Presence of typical colonies on CIN (small and smooth with a red centre and translucent rim) and on YECA (small and red fuchsia) were checked. At least two typical colonies per plate were streaked on YeCM, and these plates were incubated at 30°C for 24 hours. This step on YeCM permitted to differentiate rapidly the pathogenic *Y. enterocolitica *(red bull's-eye-like colonies) from the nonpathogenic *Y. enterocolitica *(blue-purple colonies). Confirmation and biotyping was then done by biochemical assays as described in ISO 10273:2003 standard.

## 4. Results

### 4.1. Specificity of YECA (Table 1)

The strains of *Yersinia enterocolitica* showed the expected characteristics on CIN and YeCM, that is, important growth for all the biotypes and, on CIN, small colonies, with a red centre and a translucent rim, and on YeCM, red bull's-eye-like colonies for biotype 2, 3, and 4 and blue-purple colonies for the nonpathogenic biotype 1A. 

On YECA, the three pathogenic *Y. enterocolitica *showed an important growth with numerous small and red fuchsia colonies while the nonpathogenic biotype 1A had a very small growth on YECA. Only 5 violet colonies could be observed on YECA for this biotype while streaking was done from culture containing at least 10^8^ cells per mL. YECA consequently exhibited a high inhibitor effect on the growth of the nonpathogenic biotype 1A.

On CIN, seven of the *Yersinia*-like strains grew as red colonies with translucent rim in fair number. Only *Yersinia ruckeri* was inhibited. Similar results were noted on YeCM with an inhibition of *Yersinia ruckeri* and a good growth of the other strains even though they grew as nontypical colonies. 

Absence of growth was noted also for *Yersinia ruckeri* on YECA. The other *Yersinia*-likes strains were able to growth on YECA but the number of colonies was very small indicating that YECA had to high inhibitor effect on their growth. This inhibition is useful because we saw that the colony of *Yersinia aldovae* and the colony of *Yersinia mollaretii* had similar characteristics on YECA than *Yersinia enterocolitica*, that is, small red fuchsia. These two colonies were probably observed because streaking was carried out from a culture rich in cells, around 10^8^ cells per mL. 

For the 14 non-Yersinia strains, we observed for CIN, YeCM, and YECA an absence of growth or growth but as not characteristic colonies on these media.

These results showed that it is possible on YECA to differentiate the three pathogenic *Y. enterocolitica *from the panel of strains tested in this work.

### 4.2. Sensitivity of YECA (Table 2)

The sensitivity of YECA against the four biotypes of *Yersinia enterocolitica* was compared to the one obtained on PCA, CIN, and YeCM using a 10-fold serial dilution of the four strains. 

The sensitivity of YECA was identical to those of PCA, CIN, and YeCM for the pathogenic biotypes; enumeration was possible until the dilution −8. 

But for the biotype 1A, colonies on YECA could be numerated only at the dilutions −1, −2, −3 while on PCA, CIN and YeCM, it was possible to count the colonies of this biotype until the dilution −8.

These results showed that YECA had the same sensitivity than selective and nonselective media. YECA allowed the detection of *Y. enterocolitica* strains carrying pathogenic biotype, specifically. 

### 4.3. Detection of Pathogenic Yersinia enterocolitica from Pig Tonsils

Out of the 50 tonsils, pathogenic *Y. enterocolitica* were detected on CIN and YECA, respectively, from 17 and 15 tonsils after direct streaking, from 21 and 22 tonsils after ITC-24 hours, from 28 and 28 tonsils after ITC-48 hours, and from 8 and 5 tonsils after PSB-48 hours. 

This work showed first that enrichment in ITC during 48 hours resulted in a significantly higher recovery rate of samples positive in pathogenic *Y. enterocolitica *compared to direct streaking, streaking after ITC-24 hours and streaking after PSB-48 hours. Secondly, the concordance between the results obtained from CIN and YECA is high; the same number of positive tonsils was recovered after ITC-48 hours. 

A total of 141 strains were collected on YECA and biotyped, 12 after PSB enrichment and 129 after ITC enrichment. Among the 141 strains, 135 were identified as biotype 4 (12 from PSB and 123 from ITC), two as biotype 3 and four as biotype 2. This result shows that YECA is able to detect these 3 pathogenic biotypes from naturally contaminated pig tonsils. 

## 5. Discussion

At this day, the ISO 10273-2003 standard [21] is the reference method for isolating *Yersinia enterocolitica* from foods. This method is also recommended for pig tonsils analysis [22]. However, it involves time-consuming enrichment steps followed by plating on selective media [23]. This method involves enrichment in two broths, PSB and ITC, followed by a streaking on two plates, CIN and SSDC plates, respectively. 

Cold enrichment in PSB broth was largely used for the clinical, food, and environmental samples. The major disadvantage of cold enrichment is the long period of incubation which is not appropriate for food analysis. Doyle and Hugdahl (1983) [27] showed that incubation in PSB solution during 1 to 3 days at 25°C was as effective as enrichment at 4°C during several weeks. This was recently confirmed by Van Damme et al. (2010) [20] who showed that the use of a two-day incubation period at 25°C, instead of five days, for the PSB broth, resulted in a significantly higher recovery rate of *Yersinia*.

Wauters et al. (1988) [28] developed an enrichment broth (ITC), derived from modified Rappaport, supplemented in Irgasan, Ticarcillin and potassium chlorate. The same authors indicated that enrichment in PSB broth gave better results for nonpathogenic strains, whereas enrichment in ITC broth gave better results for pathogenic strains. However, this broth proved to be effective bioserotype 4/O: 3 strains but inhibits bioserotype 2/O: 5, 27 strains [29, 30]. De Zutter et al. (1994) [31] modified ITC formula as to have a better recovery for bioserotype 2/O: 9 strains by decreasing the concentration of chloride potassium and in malachite green.

As indicated in the ISO 10273:2003 standard, *Yersinia enterocolitica *colonies on CIN agar are typically small and smooth, with a red centre and a translucent rim and, when examined with obliquely transmitted light, they are noniridescent and finely granular. On SSDC agar, *Yersinia enterocolitica *colonies are typically small and grey, with an indistinct rim, and are noniridescent and very finely granular when examined under obliquely transmitted light. 

The SSDC agar is a modified SS agar with Sodium desoxycholate and calcium chloride in order to increase its selectivity [28]. *Yersinia* tolerates strong concentrations of this salt [32]. Moreover, the calcium chloride enhances the selection of the pathogenic strains of *Y. enterocolitica*, calcium dependent, in particular, bioserotype 4/O: 3 strains [28]. This agar is largely used because of its great selectivity and of its commercial availability. However, this medium does not always allow differentiating *Yersinia* from interfering flora such as *Morganella*, *Proteus*, *Serratia,* and *Aeromonas*. 

It is Schiemann (1979) [33] who developed the medium CIN (Cefsulodin-Irgasan-Novobiocin) for the detection of *Y. enterocolitica*. The medium CIN is highly selective, especially against *Pseudomonas aeruginosa, Escherichia coli, Klebsiella pneumoniae,* and *Proteus mirabilis*. Colony morphology coupled with mannitol fermentation permitted discrimination of *Y. enterocolitica* from most of Gram-negative bacteria that can grow on this medium. Several comparative studies showed that CIN agar was the most selective medium for *Yersinia* spp. [34–36]. Micro-organisms able to ferment mannitol, like *Yersinia*, produce on CIN typical colonies after 24 hours (small and smooth colonies, with a red centre and a translucent rim). But *Citrobacter freundii, Enterobacter agglomerans,* and the species of *Aeromonas,* and *Klebsiella* produce colonies of similar morphology [37, 38]. However, users recognized that detection on CIN agar is easier since *Y. enterocolitica* has relatively more characteristic colony morphology on this medium (typical “bull's eye” appearance) compared to SSDC [20, 24]. However, Fondrevez et al. (2010) [24] recommend the use of CIN after the enrichment in ITC broth. Tested on 900 pig tonsil swabs, the authors showed that this way recovered a larger number of positive samples than with the ISO 10272:2003 procedure: 14.0% of tonsils tested positive with the new method, versus only 9.1% with the modified ISO method. 

These media, CIN and SSDC, moreover lack the ability to differentiate potentially virulent *Y. enterocolitica* from the nonpathogenic strains and other *Yersinia*. Only panel of biochemical tests (esculin hydrolysis, indole production, and fermentation of xylose and trehalose) as described in the ISO 10273:2003 method permits to identify the biotype. 

Recently, Weagant (2008) [25] has developed a chromogenic medium (YeCM) for the specific detection of pathogenic *Y. enterocolitica*. This agar contains cellobiose as the fermentable sugar, a chromogenic substrate, and selective inhibitors for suppression of colony formation of competing flora. On this medium, pathogenic *Y. enterocolitica* strains grow as red bull's-eye-like colonies while nonpathogenic *Y. enterocolitica *grows as blue-purple colonies. 

Direct use of this chromogenic agar after enrichment broth step was difficult because many nontypical colonies interfere with the visualization of the typical colonies. It is why Fondrevez et al. (2010) [24] proposed its use after the CIN step to quickly discriminate the nonpathogenic biotype from the pathogenic biotypes. While one more day is added in the detection, this method is less time consuming than the ISO 10273:2003 procedure and, with the use of YeCM, decreases the need for biochemical tests for confirmation and biotyping.

The European regulation concerning the zoonosis of food origin lies on the directive 2003/99/ that considers doing a monitoring of the principal agents responsible for food origin zoonosis, including *Yersinia enterocolitica*. In the last years, many countries showed an increasing interest in *Y. enterocolitica* epidemiology in pig production [17–19, 39, 40]. The various studies on this bacterium show also a real interest to propose other methods to detect it. Other alternative methods using PCR [8, 26] for detecting *Yersinia enterocolitica* from food or tonsil have been published. But while PCR can be useful to quickly detect suspected positive samples, only culture method enable to recover isolates which is necessary to study the spread of the bacteria from farms to humans. Recently, EFSA and the Members of the group of work *Yersinia enterocolitica* (2009) [41] proposed a national plan for monitoring *Yersinia enterocolitica* in pigs. It became necessary to have a simplified detection method which also could target directly the pathogenic biotypes responsible for human yersiniosis. 

In this paper, we tested a new selective chromogenic plate, YECA, for its specificity, and sensitivity, and we tested its capacity to detect pathogenic *Y. enterocolitica *from pig tonsils. 

YECA in this study showed a real capacity to favor the growth of the pathogenic *Y. enterocolitica* (Biotype 2, 3, and 4) with typical colonies, small, and red fuchsia. Growth of biotype 1A was much reduced with violet colonies. Absence of growth or light growth of nontypical colonies was observed for the *Yersinia*-like strains and non-*Yersinia* strains tested in this work. Moreover, numeration of pure culture of *Y. enterocolitica *strains on YECA was similar to those carried out on PCA, CIN and YeCM, except for biotype 1A for which high inhibition was observed. We observed that YECA exhibits a stronger inhibitor effect on the growth of the *Yersinia*-like strains while numerous colonies were observed on the chromogenic media YeCM developed by Weagant (2008) [25]. This is interesting because absence of other interferent bacterial flora on the media allows rapid visualization of the presence or absence of pathogenic *Y. enterocolitica *on YECA. 

When tested from naturally contaminated pig tonsils, we observed a best performance for detecting positive samples after enrichment in ITC than in PSB and we obtained similar percentage of positive samples between CIN and YECA after enrichment in ITC during 48 hours. This result is consistent with the findings of Wauters et al. (1988) [28], indicating that enrichment in PSB broth gave better results for nonpathogenic strains, whereas enrichment in ITC broth gave better results for pathogenic strains. This result is also consistent with the work of Fondrevez et al. (2010) [24], showing that use of CIN after ITC recovered a larger number of positive samples than the use of CIN after PSB and the use of SSDC after ITC. 

In this paper, isolates were confirmed as *Yersinia* and biotyped by biochemical assays as described in ISO 10273:2003 standard. This step was necessary to separate pathogenic strains from nonpathogenic strains. CIN does not differentiate biotype 1A from the pathogenic biotypes while YECA could detect directly pathogenic *Y. enterocolitica *strains. This indicates that use of YECA decreases the need for biochemical tests for confirmation and biotyping.

From naturally contaminated pig tonsils, it could be possible to isolate the three pathogenic biotypes 2, 3, and 4 on YECA after ITC enrichment; biotype 4 representing 95.7% of all isolates. In the study of Fondrevez et al. (2010) [24], the most prevalent biotype was also biotype 4 (80.2% of all isolates), followed by biotype 3 (19.4% of all isolates). But no biotype 2 strains were detected in its study probably because ITC broth and CIN plates both favour the growth of biotype 4 [31, 42]. The results of our study seem to put forward that YECA could have a better capacity for detecting biotype 2 strains than CIN but this has to be confirmed on a higher number of samples. 

In three days, it was possible to detect pathogenic *Y. enterocolitica *strains from pig tonsils when using YECA after ITC. Consequently, combination of ITC enrichment and YECA detection generates a timesaver by giving a positive test for pathogenic *Yersinia enterocolitica* in 72 hours. 

In conclusion, we have described a simplified method that efficiently detects pathogenic *Y. enterocolitica *in pig tonsils and that it is less time consuming than the ISO 10273:2003 standard. 

In this study, we used this method on pig tonsils as *Yersinia enterocolitica *becomes a preoccupation in Europe's pig production, but studies has to be carried out for testing it on foods from animal or vegetal origin. Moreover, the chromogenic media could be tested on human faecal samples to detect human yersiniosis. 

## Figures and Tables

**Table 1:** Growth and color of colonies of strains used to test the specificity of YECA media.

Strains obtained from	Name of the strains	Growth* and color of colonies on CIN plate	Growth and color of colonies on YeCM plate	Growth and color of colonies on YECA plate
*Yersinia* RNC from Pasteur Institute (Paris, France)	*Yersinia enterocolitica* biotype 2 (IP383)	+++++ red with a translucent rim	+++++ red bull's-eye-like	+++++ small red fuchsia
	*Yersinia enterocolitica* biotype 3 (IP29228)	+++++ red with a translucent rim	+++++ red bull's-eye-like	+++++ small red fuchsia
	*Yersinia enterocolitica* biotype 4 (IP134)	+++++ red with a translucent rim	+++++ red bull's-eye-like	+++++ small red fuchsia
	*Yersinia enterocolitica* biotype 1A (IP124)	+++++ red with a translucent rim	+++++ blue-purple	+ violet colonies (5)

Collection of the Pasteur Institute (Paris, France)	*Yersinia aldovae* (CIP103162)	+++++ red with translucent rim	+++++ yellow/redwith translucent rim	+ small red fuchsia (1)
	*Yersinia bercovieri *(CIP103323)	+++++ red with translucent rim	+++++ yellow/redwith translucent rim	++ yellow/small red fuchsia
	*Yersinia frederiksenii *(CIP80.29)	+++++ red with translucent rim	+++++ blue to green	++ green/small red fuchsia
	*Yersinia kristensenii *(CIP80.30)	+++++ red with translucent rim	++++redwith translucent rim	++ pink//small red fuchsia
	*Yersinia massiliensis *(CIP109351)	+++++ red with translucent rim	+++++ green	++ green/small red fuchsia
	*Yersinia mollaretii *(CIP103324)	+++++ red with translucent rim	+++++ yellow/redwith translucent rim	+ small red fuchsia (1)
	*Yersinia rohdei *(CIP103163)	+++++ red with translucent rim	+++++ yellow/redwith translucent rim	+ pink (1)
	*Yersinia ruckeri *(CIP82.80)	No growth	No growth	No growth

Collection of the Pasteur Institute (Paris, France)	*Salmonella* Typhimurium (CIP55.43)	No growth	No growth	No growth
	*Campylobacter jejuni* (CIP70.2)	No growth	No growth	No growth
	*Enterococcus faecalis* (CIP55/42)	No growth	No growth	No growth
	*Lactobacillus plantarum * (CIP103151)	No growth	No growth	No growth
	*Pseudomonas fluorescens* (CIP525)	+++++ yellow	+++++ yellow	+ pink
	*Brochothrix thermosphacta* (CIP103251)	No growth	No growth	No growth

Field strains from Anses collection	*Listeria monocytogen*es	No growth	No growth	No growth
	*Escherichia coli*	No growth	No growth	No growth
	*Staphylococcus aureus*	No growth	No growth	No growth
	*Klebsiella* sp.	No growth	No growth	No growth
	*Proteus mirabilis*	No growth	No growth	No growth

Strains from Fondrevez et al., (2010)	*Morganella morganii*	+++++ yellow	+++++ yellow	++ yellow/pink
	*Pseudomonas sp.*	+++++ yellow	+++++ yellow	+ pink
	*Serratia liquefaciens*	+++++ pink with translucent rim	+++++ green	++++ green/blue/pink

*Growth was measured from no growth (absence of colonies) to 5 +++++ (important culture with numerous colonies).

**Table 2:** Sensibility of YECA compared to PCA, CIN and YeCM against the four biotypes of *Yersinia enterocolitica*.

Dilution of the culture	Biotype 1A (IP124)				Biotype 2 (IP383)				Biotype 3 (IP29228)				Biotype 4 (IP134)			
	PCA	CIN	YeCM	YECA	PCA	CIN	YeCM	YECA	PCA	CIN	YeCM	YECA	PCA	CIN	YeCM	YECA

−1				150 VC				NN				NN				NN
−2				32 VC				NN				NN				NN
−3				2 VC				NN				NN				NN
−4				0				NN				NN				NN
−5	>200	>200	>150	0	>300	>300	>300	>300 RF	>300	>250	>250	>400 RF	>300	>300	>300	>300 RF
−6	53	39	46	0	34	53	50	49 RF	86	101	82	91 RF	70	77	78	63 RF
−7	3	3	2	0	3	5	6	6 RF	12	11	9	12 RF	5	7	8	10 RF
−8	1	1	1	0	0	1	1	1 RF	0	0	1	2 RF	0	2	0	1 RF
−9	0	0	0	0	0	0	0	0	0	1	0	0	0	0	0	0
−10	0	0	0	0	0	0	0	0	0	0	0	0	0	0	0	0

VC: violet colonies; RF: red fuchsia colonies; NN: nonnumerable.

## References

[1] Savin C, Carniel E (2008). Les diarrhées d’origine bactérienne : le cas de *Yersinia enterocolitica*. *Revue Francophone des Laboratoires*.

[2] EFSA (2010). The community summary report on trends and sources of zoonoses, zoonotic agents and food-borne outbreaks in the European Union in 2008. *EFSA Journal*.

[3] EFSA (2009). Community report on trends & sources of zoonoses and zoonotic agents in the EU in 2007. *EFSA Journal*.

[4] Bottone EJ (1999). *Yersinia enterocolitica*: overview and epidemiologic correlates. *Microbes and Infection*.

[5] Kapperud G, Olsvik O (1982). Isolation of enterotoxigenic *Yersinia enterocolitica* from birds in Norway. *Journal of Wildlife Diseases*.

[6] Lindblad M, Lindmark H, Lambertz ST, Lindqvist R (2006). Microbiological baseline study of broiler chickens at Swedish slaughterhousess. *Journal of Food Protection*.

[7] Bucher M, Meyer C, Grotzbach B, Wacheck S, Stolle A, Fredriksson-Ahomaa M (2008). Epidemiological data on pathogenic *Yersinia enterocolitica* in Southern Germany during 2000–2006. *Foodborne Pathogens and Disease*.

[8] Lambertz ST, Nilsson C, Hallanvuo S, Lindblad M (2008). Real-time PCR method for detection of pathogenic *Yersinia enterocolitica* in Food. *Applied and Environmental Microbiology*.

[9] Bonardi S, Paris A, Bassi L (2010). Detection, semiquantitative enumeration, and antimicrobial susceptibility of *Yersinia enterocolitica* in pork and chicken meats in Italy. *Journal of Food Protection*.

[10] Ostroff SM, Kapperud G, Hutwagner LC (1994). Sources of sporadic *Yersinia enterocolitica* infections in Norway: a prospective case-control study. *Epidemiology and Infection*.

[11] Fosse J, Seegers H, Magras C (2009). Prevalence and risk factors for bacterial food-borne zoonotic hazards in slaughter pigs: a review. *Zoonoses and Public Health*.

[12] Thibodeau V, Frost EH, Chénier S, Quessy S (1999). Presence of *Yersinia enterocolitica* in tissues of orally-inoculated pigs and the tonsils and feces of pigs at slaughter. *Canadian Journal of Veterinary Research*.

[13] Nesbakken T, Eckner K, Hoidal HK, Rotterud OJ (2003). Occurrence of *Yersinia enterocolitica* and *Campylobacter* spp. in slaughter pigs and consequences for meat inspection, slaughtering, and dressing procedures. *International Journal of Food Microbiology*.

[14] Skjerve E, Lium B, Nielsen B, Nesbakken T (1998). Control of *Yersinia enterocolitica* in pigs at herd level. *International Journal of Food Microbiology*.

[15] Fredriksson-Ahomaa M, Björkroth J, Hielm S, Korkeala H (2000). Prevalence and characterization of pathogenic *Yersinia enterocolitica* in pig tonsils from different slaughterhouses. *Food Microbiology*.

[16] Bonardi S, Brindani F, Pizzin G (2003). Detection of *Salmonella* spp., *Yersinia enterocolitica* and verocytotoxin-producing *Escherichia coli* O157 in pigs at slaughter in Italy. *International Journal of Food Microbiology*.

[17] Gürtler M, Alter T, Kasimir S, Linnebur M, Fehlhaber K (2005). Prevalence of *Yersinia enterocolitica* in fattening pigs. *Journal of Food Protection*.

[18] Kechagia N, Nicolaou C, Ioannidou V (2007). Detection of chromosomal and plasmid—encoded virulence determinants in *Yersinia enterocolitica* and other *Yersinia* spp. isolated from food animals in Greece. *International Journal of Food Microbiology*.

[19] Laukkanen R, Martinez PO, Siekkinen KM, Ranta J, Maijala R, Korkeala H (2009). Contamination of carcasses with human pathogenic *Yersinia enterocolitica* 4/O:3 originates from pigs infected on farms. *Foodborne Pathogens and Disease*.

[20] Van Damme I, Habib I, De Zutter L (2010). *Yersinia enterocolitica* in slaughter pig tonsils: enumeration and detection by enrichment versus direct plating culture. *Food Microbiology*.

[21] ISO 10273 (2003). *Microbiology of Food and Animal Feeding Stuffs—Horizontal Method for the Detection of Presumptive Pathogenic Yersinia enterocolitica (ISO 10273:2003)*.

[22] EFSA (2007). Scientific opinion of the panel on BIOHAZ on a request from EFSA on monitoring and identification of human enteropathogenic *Yersinia* spp. *The European Food Safety Authority *.

[23] De Boer E (1992). Isolation of *Yersinia enterocolitica* from foods. *International Journal of Food Microbiology*.

[24] Fondrevez M, Labbé A, Houard E (2010). Simplified method for detecting pathogenic *Yersinia enterocolitica* in slaughtered pig tonsils. *Journal of Microbiological Methods*.

[25] Weagant SD (2008). A new chromogenic agar medium for detection of potentially virulent *Yersinia enterocolitica*. *Journal of Microbiological Methods*.

[26] Fredriksson-Ahomaa M, Wacheck S, Koenig M, Stolle A, Stephan R (2009). Prevalence of pathogenic *Yersinia enterocolitica* and *Yersinia pseudotuberculosis* pseudotuberculosis in wild boars in Switzerland. *International Journal of Food Microbiology*.

[27] Doyle MP, Hugdahl MB (1983). Improved procedure for recovery of *Yersinia enterocolitica* from meats. *Applied and Environmental Microbiology*.

[28] Wauters G, Goossens V, Janssens M, Vandepitte J (1988). New enrichment method for isolation of pathogenic *Yersinia enterocolitica* serogroup O:3 from pork. *Applied and Environmental Microbiology*.

[29] Kwaga J, Iversen JO, Saunders JR (1990). Comparison of two enrichment protocols for the detectionof *Yersinia* in slaughtered pigs and pork products. *Journal of Food Protection*.

[30] De Boer E, Nouws JFM (1991). Slaughter pigs and pork as a source of human pathogenic *Yersinia enterocolitica*. *International Journal of Food Microbiology*.

[31] De Zutter L, Le Mort L, Janssens M, Wauters G (1994). Short-comings of irgasan titarcillin chlorate broth for the enrichment of *Yersinia enterocolitica* biotype 2, serotype 9 from meat. *International Journal of Food Microbiology*.

[32] Leclercq A Le genre *Yersinia* et son incidence dans le domaine alimentaire.

[33] Schiemann DA (1979). Synthesis of a selective agar medium for *Yersinia enterocolitica*. *Canadian Journal of Microbiology*.

[34] Schiemann  DA (1983). Comparison of enrichment and plating media for recovery of virulent strains of *Yersinia enterocolitica* from inoculated beef stew. *Journal of Food Protection*.

[35] Walker SJ, Gilmour A (1986). The incidence of *Yersinia enterocolitica* and *Yersinia enterocolitica*-like organismsin raw and pasteurised milk in Northern Ireland. *The Journal of Applied Bacteriology*.

[36] Cox NA, Bailey JS, Del Corral F, Shotts EB (1990). Comparison of enrichment and plating media for isolation of *Yersinia*. *Poultry Science*.

[37] Devenish JA, Schiemann DA (1981). An abbreviated scheme for identification of *Yersinia enterocolitica* isolated from food enrichment on CIN (cefsulodin-irgasannovobiocin) agar. *Canadian Journal of Microbiology*.

[38] Harmon MC, Yu CL, Swaminathan B An evaluation of selective differential plating media forthe isolation of *Yersinia enterocolitica* from experimentally inoculated fresh ground pork homogenate. *Journal of Food Science*.

[39] Von Altrock A, Louis AL, Rosler U (2006). The bacteriological and serological prevalence of *Campylobacter* spp and *Yersinia enterocolitica* fattening pig herds in Lower Saxony. *Berliner und Munchener Tierarztliche Wochenschrift*.

[40] Milnes AS, Stewart I, Clifton-Hadley FA (2008). Intestinal carriage of verocytotoxigenic *Escherichia coli*, *Salmonella*, thermophilic *Campylobacter* and *Yersinia enterocolitica*, in cattle, sheep and pigs at slaughter in Great Britain during 2003. *Epidemiology and Infection*.

[41] EFSA and the Members of the group of work Yersinia enterocolitica (2009). Technical specifications for harmonized national surveys on *Yersinia enterocolitica* in slaughter pigs. *EFSA Journal*.

[42] Schiemann DA (1982). Development of a two-step enrichment procedure for recovery of *Yersinia enterocolitica* from food. *Applied and Environmental Microbiology*.

